# Sense of coherence and substance use in adults: a systematic review and meta-analysis

**DOI:** 10.1590/0102-311XEN141323

**Published:** 2024-09-20

**Authors:** Júlia Freire Danigno, Mariane da Silva Dias, Bernardo Lessa Horta

**Affiliations:** 1 Universidade Federal de Pelotas, Pelotas, Brasil.; 2 Universidade Federal do Rio Grande, Rio Grande, Brasil.

**Keywords:** Sense of Coherence, Substance Use, Smoking, Illicit Drug, Alcohol Drinking, Senso de Coerência, Uso de Substâncias, Fumar, Droga Ilícita, Consumo de Bebidas Alcoólicas, Sentido de Coherencia, Uso de Sustancias, Fumar, Droga Ilícita, Consumo de Bebidas Alcohólicas

## Abstract

This study systematically reviews the evidence on the association between sense of coherence (SOC) and substance use during adulthood. Two researchers conducted independent literature searches on the PubMed, LILACS, PsycINFO and Web of Science databases. Original articles assessing SOC and substance use in adults (age > 19 years) were included. Two reviewers independently assessed studies in two phases - initially by reading the title/abstract, then the full text. Discrepancies were resolved by a third reviewer. Estimates were pooled using random-effects models. Bibliographic search identified 21 studies on the association between SOC and substance use in adults. Studies (n = 11) that assessed the association with tobacco smoking found a 0.92 (95%CI: 0.82; 1.01, very low degree of certainty) odds of smoking among those with a high SOC; the association was not modified by age. Individuals with a strong SOC had lower odds of using alcohol (pooled effect: OR = 0.70, 95%CI: 0.50; 0.90, very low degree of certainty); adjustment for confounding variables decreased the magnitude of the association (pooled OR = 0.89, 95%CI: 0.80; 0.98). This systematic review and meta-analysis suggests that a strong SOC protects against substance use among adults regardless of age, with practical implications for preventive interventions and tailored strategies aimed at high-risk individuals. Longitudinal studies are needed to understand the impact of SOC on substance use. Examining interactions with socioeconomic factors and including diverse populations would enhance generalizability.

## Abstract (pt)

O objetivo deste estudo foi fazer uma revisão sistemática das evidências sobre a associação entre senso de coerência (SOC) e uso de substâncias na vida adulta. Dois pesquisadores buscaram, independentemente, as bases de dados PubMed, LILACS, PsycINFO e Web of Science. Foram incluídos artigos originais que avaliaram o SOC e o uso de substâncias em adultos (idade > 19 anos). Dois revisores avaliaram, independentemente, os estudos em duas fases - inicialmente com base no título/resumo e, em seguida, no texto completo. Discrepâncias foram resolvidas por um terceiro revisor. Modelos de efeitos aleatórios foram usados para agrupar as estimativas. Identificamos 21 estudos que investigaram a associação entre SOC e uso de substâncias em adultos. Entre os 11 estudos que avaliaram a associação com o tabagismo, a chance de fumar foi de 0,92 (IC95%: 0,82; 1,01, grau de confiança muito baixo) entre aqueles com alto SOC, e a associação não foi modificada pela idade. Indivíduos com forte SOC tiveram menores chances de usar álcool (efeito combinado: OR = 0,70, IC95%: 0,50; 0,90, grau de confiança muito baixo), e o ajuste para confusão diminuiu a magnitude da associação (OR combinado = 0,89, IC95%: 0.80; 0.98). Esta revisão sistemática e metanálise sugere que um forte SOC protege contra o uso de substâncias entre adultos, independentemente da idade, com implicações práticas para intervenções preventivas e estratégias personalizadas, especialmente para indivíduos de alto risco. Estudos longitudinais são necessários para entender o impacto do SOC no uso de substâncias. Examinar as interações com fatores socioeconômicos e incluir diversas populações aumentaria a generalização.

## Abstract (es)

Este estudio realiza una revisión sistemática sobre la asociación entre sentido de coherencia (SOC) y uso de sustancias en la edad adulta. Dos investigadores realizaron búsquedas de forma independiente en las bases de datos PubMed, LILACS, PsycINFO y Web of Science. Se incluyeron artículos originales que evaluaron el SOC y el uso de sustancias por adultos (edad > 19 años). Dos evaluadores examinaron de forma independiente los estudios en dos etapas: primero, el título/resumen; y, después, el texto completo. Las discrepancias fueron resueltas por un tercer evaluador. Se utilizaron modelos de efectos aleatorios para agrupar las estimaciones. Se identificaron 21 estudios sobre la asociación entre SOC y uso de sustancias por adultos. De 11 estudios que evaluaron la asociación con el tabaquismo, la propensión al tabaquismo fue de 0,92 (IC95%: 0,82; 1,01, grado de confianza muy bajo) entre las personas con alto SOC; y la asociación no sufrió cambios según la edad. Las personas con alto SOC tenían menor propensión a consumir alcohol (efecto combinado: OR = 0,70, IC95%: 0,50; 0,90, grado de confianza muy bajo), y el ajuste por confusión disminuyó la intensidad de la asociación (OR combinado = 0,89, IC95%: 0,80; 0,98). Esta revisión sistemática y metanálisis muestra que un alto SOC protege contra el uso de sustancias por adultos, independientemente de la edad, con implicaciones prácticas para las intervenciones de prevención y las estrategias personalizadas, especialmente para las personas con alto riesgo. Se necesitan estudios longitudinales para comprender el impacto del SOC en el uso de sustancias. Analizar las interacciones con los factores socioeconómicos e incluir otras poblaciones podría aumentar la generalización.

## Introduction

Sense of coherence (SOC) is a concept introduced by Aaron Antonovsky’s salutogenesis framework. It aims to understand the contributing factors in the development of health and to explain how individuals can manage their lives despite adverse conditions ^1^ ^,^ ^2^. SOC comprises three components: comprehensibility (ability to understand an event), manageability (perceived potential to manipulate or resolve the event), and meaningfulness (significance attributed to this event) ^2^.

A strong SOC empowers individuals to mobilize internal and external resources to effectively cope with stressors and manage tension, thereby promoting and maintaining their health ^1^. Individuals with a strong SOC would be more efficient in creating coping mechanisms and strategies to maintain health in unfavorable situations ^2^. Urakawa & Yokoyama ^3^ observed that SOC is negatively associated with stress levels and positively correlated with the ability to cope with stress. This reduction in stress would positively influence health-related behaviors, contributing to maintain a positive health status.

Evidence suggests that SOC is associated with various health-related behaviors like tobacco smoking ^4^, alcohol intake ^5^, and illicit drug use ^6^. These behaviors are associated with the development of noncommunicable diseases (NCDs) such as cardiovascular diseases, cancer, diabetes, and chronic respiratory conditions, which account for two-thirds of the overall burden of disease in low- and middle-income countries ^7^ ^,^ ^8^. In 2019, smoking resulted in 8.71 million attributable deaths (15.4% of all deaths), alcohol use led to 2.07 million attributable deaths among men and 0.40 million among women, and drug use contributed to 0.45 million attributable deaths ^8^. SOC would also have an impact in the development of NCDs through health-related behaviors.

It is crucial to recognize and critically assess the methodological limitations within research. Many studies assessed the association between SOC and health-related behaviors without controlling for potential confounding factors. Failure to address known confounders can overestimate the association. For example, low socioeconomic status is associated with lower SOC scores and less favorable behavioral habits. Consequently, analyses that fail to control for confounding by socioeconomic status are susceptible to residual confounding which would overestimate the magnitude of the association.

Currently, only the study by da-Silva-Domingues et al. ^9^ has reviewed the relation between SOC and substance use, but as part of a broader analysis that focused on health behaviors such as eating habits, time spent on computers, rest periods, as well as smoking, alcohol consumption, and oral health care. However, it specifically evaluates the association between SOC and substance use (tobacco, alcohol, and illicit drugs). It is crucial to recognize how SOC influences both general health behaviors and specifically risky behaviors like substance use, given their significant impact on public health and the development of chronic diseases. While da-Silva-Domingues et al.’s study ^9^ assessed individuals aged 12 to 30 years, our research seeks to bridge a gap by concentrating on the adult population as defined by the World Health Organization (WHO). By identifying heterogeneity sources and conducting a meta-analysis, the present study intends to offer insights beyond the existing literature which can guide future research and interventions aimed at reducing harmful substance use among adults.

In short, this study reviewed the literature on the association between substance use (tobacco, alcohol, and illicit drugs) and SOC, while exploring sources of heterogeneity.

## Methods

This systematic review was registered on PROSPERO (protocol n. CRD42023402776) and conducted according to PRISMA (*Preferred Reporting Items for Systematic Reviews and Meta-Analyses*) and AMSTAR2 (*A MeaSurement Tool to Assess systematic Reviews*) guidelines (Supplementary Material - Boxes 1 and 2; https://cadernos.ensp.fiocruz.br/static//arquivo/suppl-e00141323_3035.pdf). We formulated the following research question: “What is the association between sense of coherence and substance use in adults?” (P = adults; I = high SOC; C = low SOC; O = substance use outcomes).

### Search protocol and selection criteria

Bibliographic search was conducted in March 2023 on the PubMed, LILACS, Web of Science, and PsycINFO databases. Search strategies combined the terms for SOC (“sense of coherence” OR salutogen* OR “general resistance resources”) with the following terms for each of the studied outcomes:

Smoking, cigarette smoking, tobacco, and tobacco use disorder;
Alcohol, alcoholism, and alcohol drinking;
Substance use, substance-related disorders, and substance abuse;
Illicit drugs, cocaine, crack, cannabis, amphetamine, and narcotic.

Supplementary Material - Table S1 (https://cadernos.ensp.fiocruz.br/static//arquivo/suppl-e00141323_3035.pdf) shows the search strategy and the number of studies identified.

Inclusion criteria consisted of original articles that evaluated the association of SOC with at least one measure of substance use among adult participants (age > 19 years). We set no restrictions on language, publication date, or the SOC measurement scale used. Papers involving animals, research protocols, editorials, comments, and those with insufficient data were excluded.

Additionally, we searched for grey literature in the Google Scholar, CAPES Portal of Theses and Dissertations, and ProQuest databases.

### Data extraction (selection and coding)

Study selection was performed in two phases. First, two independent reviewers evaluated the title and abstract of each identified study. Articles considered as possibly eligible for inclusion in the review were retrieved and read in full. Discrepancies between the reviewers regarding the inclusion or exclusion of a paper were resolved by a third reviewer.

Last name of the first author, year of publication, country where the study was conducted, sample size, age and gender of the studied population, study design, scale used for substance use measurement, categorization of substance use, scale used for SOC assessment, SOC categorization, control for confounding factors, and effect measurement along with its 95% confidence interval (95%CI) were extracted. Two independent reviewers extracted the data using a Microsoft Excel spreadsheet (https://products.office.com/). Any discrepancies between reviewers were resolved through consensus or consultation with a third reviewer. Subsequently, the extracted data were transferred to Stata software (https://www.stata.com).

Methodological quality of the included studies was evaluated using the *Risk of Bias in Non-randomized Studies of Exposures* scale (ROBINS-e) ^10^, recommended by the Cochrane Collaboration for assessing effectiveness and safety in nonrandomized intervention trials. This instrument has seven domains of bias categorized by the timing of occurrence: pre-intervention (confounding and selection bias in participant enrollment), at intervention (bias in classifying interventions), and post-intervention (deviations from intended interventions, missing data bias, outcome measurement bias, and bias in selection of reported results). Assessment classified items as low, moderate, severe, or critical risk of bias, or as having no information, following the *Cochrane Handbook for Systematic Reviews of Interventions* ^11^.

### Data synthesis and analysis plan

For inclusion in the meta-analysis, the studies had to report a crude or adjusted measure of the association between SOC and substance use. We considered the following reported measures: mean values of SOC for both users and non-users, linear regression coefficients, odds ratios (OR), and prevalence ratios of substance use in different SOC categories along with their corresponding 95%CI or standard errors.

Pooled measure of association was calculated using a random effects model. We conducted two distinct meta-analyses - one focused on substance use outcomes; the other focused on the mean SOC - using Stata. The analyses were stratified according to the type of substance, follow-up rate, sample size, age group, confounding variables (socioeconomic status), study setting (America, Asia, Europe), SOC categorization, smoking and alcohol.

Heterogeneity between studies was assessed using the Q-test and I-square. Estimates were pooled using a random-effects model. Publication bias was assessed using a funnel plot and Egger’s test. We also stratified the analysis according to sample size to further evaluate the impact of publication bias on the estimates.

Certainty of evidence was evaluated using the GRADEpro software (https://www.gradepro.org/). The GRADE (*Grading of Recommendations Assessment, Development, and Evaluation*) system categorizes evidence quality into four levels - high, moderate, low, and very low - based on considerations such as study design limitations, indirect evidence, inconsistency of results, imprecision of results, and probability of publication bias.

## Results

### Characteristics of the included studies

**Figure 1:**
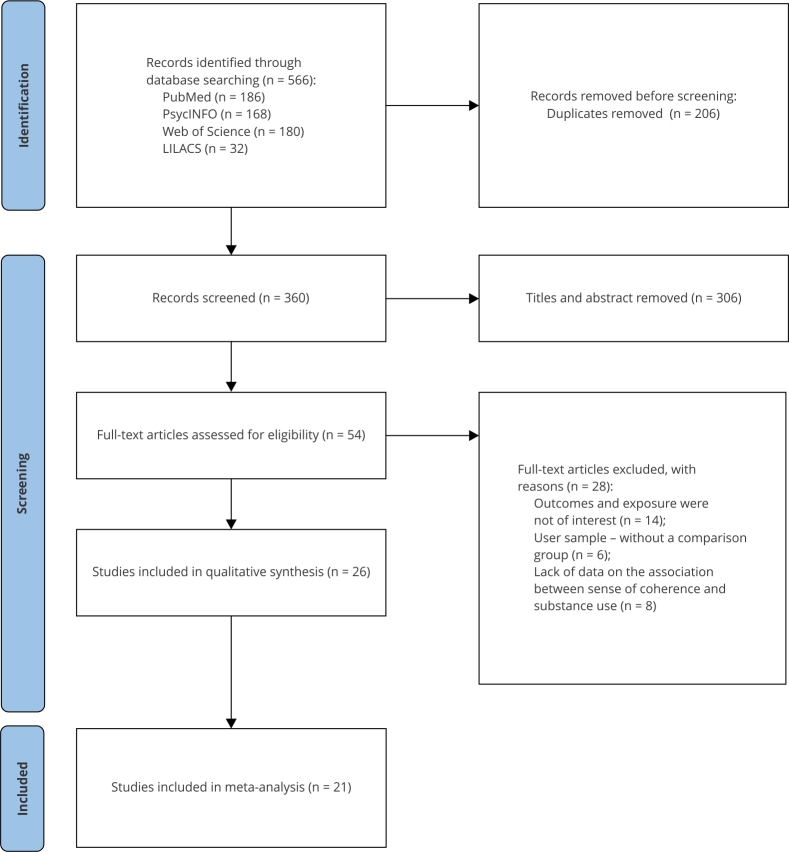
Flowchart of study selection.

Database search identified a total of 566 records (Figure 1). After removing duplicates (n = 206 articles), 360 titles and abstracts were read resulting in 54 studies selected for full text review. In the end, the review sample included 21 articles. Exclusion criteria consisted of articles with outcomes and exposure that were not of interest (n = 14), user sample (n = 6), and lack of data on the association between SOC and substance use (n = 8). Supplementary Material - Box S3 (https://cadernos.ensp.fiocruz.br/static//arquivo/suppl-e00141323_3035.pdf) shows the references excluded after full text review and the respective reason. An additional manual search was performed on the references of the 21 selected articles, but we identified no additional study.

### Characterization and qualitative synthesis of the selected studies

Table 1 presents the main characteristics of the included studies. Most were cross-sectional (n = 14) and longitudinal (n = 6), conducted in European countries (n = 17) and published from 1987 to 2022. Sample size varied considerably, ranging from 120 to 40,674 participants. Participant age also exhibited a wide range, from 15 to 88 years. SOC was assessed by different instruments, namely: SOC-L9 (n = 2), SOC-7 (n = 1), SOC-3 (n = 3), SOC-29 (n = 7), and SOC-12 (n = 2).

**Table 1:** Characteristics of the selected studies: odds ratios (OR) for strong sense of coherence (SOC) and their effect on substance use (smoking, alcohol, illicit drugs) in 14 studies and mean difference in SOC regarding substance use (smoking, alcohol) in seven studies.

Study/Country (Year)	Study design	Sample/Follow-up rate (%)	Mean age (in years)	SOC	Categorization of SOC	Substance use	Scale of substance use	Categorization of substance use	Effect measures (95%CI) *	Control for confounding
Neuner et al. ^16^ Germany (2006)	Prospective	2,056 30.2%	34 ± 12	SOC-L9	Quartiles: strongest vs. weakest	Alcohol	AUDIT score	Hazardous alcohol consumption (yes/no). Yes, in men: AUDIT 8-40 points; in women: AUDIT 5-40 points)	OR = 0.51 (0.36; 0.72)	Age, gender, income, education, additional substance use
						Smoking	Structured self-designed questionnaire	Smokers (current)/ex and non-smokers	OR = 0.59 (0.45; 0.78)	
						Drugs	Structured self-designed questionnaire	No/Yes. Yes: use of illicit drugs at least 1 to 3 times within the last 12 months	OR = 0.46 (0.33; 0.66)	
Antonovsky et al. ^17^ Israel (1987)	Cross-sectional	120 (male) 83.64%	41.1	SOC-7	Dichotomous: strong vs. weak	Alcohol	Structured self-designed questionnaire	Drinkers/Non-drinkers. Drinkers: drink once to several times per day	OR = 0.46 (0.18; 1.18)	No
Tobamidanik & Zabkiewicz ^18^ United States (2009)	Cross-sectional	4,630	62.4 ± 19	SOC-3	Terciles: strongest vs. weakest	Alcohol	Alcohol dependence - DSM-IV	Drinkers/Non-drinkers. Drinkers: 5 or more drinks consumed at least once per week	OR = 0.30 (0.24; 0.37)	Age, gender, income, education, additional substance use
Wainwright et al. ^19^ United Kingdom (2007)	Cross-sectional	18,287 87.40%	41.0-80.0	SOC-3	Dichotomous: strong vs. weak	Smoking	Structured self-designed questionnaire	Smokers (current)/ex and non-smokers	OR = 0.74 (0.63; 0.86)	Age, gender, income, education
Morita et al. ^5^ Japan (2014)	Cross-sectional	167 90%	41.9 ± 9.8	SOC-29	Terciles: strongest vs. weakest	Smoking Alcohol	Lifestyle-related questionnaire	Smokers (current)/ex and non-smokers	OR = 1.19 (0.43; 3.34)	Age, gender, income, social support
							Lifestyle-related questionnaire	Drinkers/Non-drinkers. Drinkers: drinking more than 1 gou ** per day	OR = 0.78 (0.33; 1.85)	
Savolainen et al. ^20^ Finland (2009)	Cross-sectional	8,028 88%	41.8 ± 10.6	SOC-12	Quintiles: strongest vs. weakest	Smoking	Structured self-designed questionnaire	Smokers (current)/ex and non-smokers. Smokers: regular or occasional smokers	OR = 0.75 (0.61; 0.91)	Age, gender, education
Silarova et al. ^4^ Slovakia (2014)	Cross-sectional	179 60.1%	58.32 ± 6.54	SOC-13	Dichotomous: strong vs. weak	Alcohol	*European Health and Behaviour Survey*	Drinkers/Non-drinkers. Drinkers: occasional and regular	OR = 1.01 (0.97; 1.05)	Age, gender, income
						Smoking	*European Health and Behaviour Survey*		OR = 1.06 (1.00; 1.13)	
van Loon et al. ^21^ The Netherlands (2001)	Longitudinal	1,431 women 54%	42.6 ± 10.9	SOC-3	Dichotomous: strong vs. weak	Smoking	*The Health and Life Experiences Questionary*	Smokers (current) and ex/never	OR = 1.16 (0.98; 1.36)	Age
		1,083 men							OR = 1.05 (0.88; 1.25)	
Saade & Marchand ^22^ Canada (2013)	Longitudinal	7,338 81%	43.82 ± 10.16	SOC-13	Numerical	Alcohol	Canadian norms	Alcohol misuse. Yes: man drinks more than 14 drinks per week or when a woman drinks more than 9 drinks per week	OR = 0.99 (0.98; 1.00)	Age, gender, income, education, marital status, social support
Von Ah et al. ^23^ United States (2005)	Cross-sectional	161 40%	19.6 ± 4.09	SOC-29	Numerical	Smoking	4-item tobacco self-efficacy questionnaire	Smokers (current)/ex and non-smokers. Current smoker: an individual who smoked a whole cigarette within the last 30 days	OR = 1.01 (0.97; 1.04)	No
Ristkari et al. ^6^ Finland (2005)	Longitudinal	2,314 78.7%	67 ± 11	SOC-13	Quartiles: strongest vs. weakest	Smoking	Structured self-designed questionnaire	Smokers (current) and ex/never. Smokers: smoked during the last 6 months	OR = 0.99 (0.78; 1.26)	No
						Alcohol	Structured self-designed questionnaire	Drinker/Non-drinkers. Drunkers: drunk during the last 6 months	OR = 0.69 (0.49-0.87)	
						Drugs	Structured self-designed questionnaire	Yes/No. Yes: drugs during the last 6 months	OR = 0.18 (0.10; 0.33)	
Larm et al. ^24^ Sweden (2016)	Cross-sectional	40,674 59.2%	53.8 ± 17.9	SOC-13	Terciles: strongest vs. weakest	Alcohol	AUDIT-C	Hazardous alcohol consumption. Yes, in men: AUDIT 8-40 points; in women: AUDIT 6-40 points)	OR = 0.48 (0.36; 0.64)	Yes
Thomas et al. ^25^ Sweden (2020)	Cross-sectional	1,007 62.5%	57 ± 7.2	SOC-29	Numerical	Alcohol	Structured self-designed questionnaire	Hazardous alcohol consumption. Drinking more than 9 standard glasses per week for women and more than 14 glasses per week for men, and/or reporting drinking 4 or more standard glasses for women and 5 or more glasses for men on a typical day when drinking	OR = 0.79 (0.64; 0.96)	Age, sex, education, income
						Smoking	Structured self-designed questionnaire	Smokers (current)/ex and non-smokers	OR = 0.84 (0.71; 1.00)	
Poppius et al. ^26^ Finland (1999)	Longitudinal	4,405 73%	40-55	SOC-29	Terciles: strongest vs. weakest	Smoking	Structured self-designed questionnaire	Smokers (current)/ex and non-smokers	OR = 0.96 (0.82; 1.12)	Age
Gajdosova et al. ^27^ Slovakia (2009)	Cross-sectional	830 94.1%	20.5 ± 1.4	SOC-13	Numerical	Smoking	Structured self-designed questionnaire	Smokers (current)/ex and non-smokers	β: -0.61 (-2.06; 0.84)	No
Ahlstrand et al. ^28^ Sweden (2022)	Cross-sectional	851 37.3%	28	SOC-13	Numerical	Smoking	Structured self-designed questionnaire	Smokers (current)/ex and non-smokers. No: no or rarely	β: -3.33 (-5.63; -1.03)	Gender, additional substance use
						Alcohol	Structured self-designed questionnaire	Smokers (current)/ex and non-smokers. Current: > once per month	β -1.76 (-3.37-0.15)	
Luszczynska ^29^ Poland (2002)	Cross-sectional	83 women 100%	35.6 ± 9.0	SOC-29	Terciles: strongest vs. weakest	Smoking	Structured self-designed questionnaire	Cigarettes daily	β: -4.50 (-10.87; 1.86)	No
Vilela & Alisson ^30^ Canada (2010)	Cross-sectional	162	25-88	SOC-13	Numerical	Smoking	Structured self-designed questionnaire	Smokers (current) and ex/non-Smokers	β: -4.40 (-13.73; 4.91)	Age, education, marital status
						Alcohol	Structured self-designed questionnaire	Smokers (current) and ex/non-smokers	β: 2.30 (-10.47; 15.06)	
Igna et al. ^31^ Sweden (2008)	Cross-sectional	841	59.4 ± 8.1	SOC-13	Numerical	Smoking	Structured self-designed questionnaire	Smokers (current) and ex/non-smokers	β: -3.20 (-4.79; -1.61)	
Verešová & Gatial ^32^ Romania (2010)	Cross-sectional	158	19-25	SOC-29	Numerical	Alcohol	No information	Smokers (current)/ex and non-smokers	β: -38.0 (-47.8; -28.8)	No
Kouvonen et al. ^33^ Finland (2008)	Longitudinal	313 5.7%	36-82	SOC-13	Terciles: strongest vs. weakest	Alcohol	Structured self-designed questionnaire	Hazardous alcohol consumption. Excessive drinking leading intoxication twice or more per month vs. less than twice per month	β: -4.24 (-4.27; -4.21)	Age, education, marital status, additional substance use

95%CI: 95% confidence interval; AUDIT: *Alcohol Use Disorders Identification Test*; DSM-IV: *Diagnostic and Statistical Manual of Mental Disorders* - 4th edition.

* OR: odds ratios for sense of coherence (strong) and their effect on substance use (smoking, alcohol, illicit drugs), β: mean difference in sense of coherence regarding substance use (smoking, alcohol);

** Traditional Japanese unit of alcohol beverage.

### Risk of bias assessment

Quality assessment of the risk of bias was predominantly moderate (n = 10), eight studies presented critical risk and four low risk of bias (Box 1). Missing data was the main criteria contributing to moderate or critical risk of bias.

Table 2 summarizes the certainty of the evidence for the outcomes included in the meta-analysis.

**Box 1:** Risk of bias assessment in nonrandomized studies (ROBINS-e - *Risk of Bias in Non-randomized Studies of Exposures scale*).

STUDY	BIAS DUE TO CONFOUNDING	BIAS IN SELECTION OF PARTICIPANTS INTO THE STUDY	BIAS IN CLASSIFICATION OF INTERVENTIONS	BIAS DUE TO DEVIATIONS FROM INTENDED INTERVENTIONS	BIAS DUE TO MISSING DATA	BIAS IN MEASUREMENT OF OUTCOMES	BIAS IN SELECTION OF THE REPORTED RESULT	OVERALL BIAS
Antonovsky et al. ^17^	Serious	Serious	Low	Moderate	Moderate	Moderate	Low	Serious
Ahlstrand et al. ^28^	Moderate	Low	Low	Low	Moderate	Low	Low	Moderate
Gajdosova et al. ^27^	Serious	Serious	Low	Low	Low	Low	Low	Serious
Igna et al. ^31^	Serious	Low	Low	Low	NI	Low	Low	Serious
Kouvonen et al. ^33^	Low	Low	Low	Low	Moderate	Low	Low	Moderate
Larm et al. ^24^	Low	Low	Low	Low	Low	Low	Low	Low
Luszczynska ^29^	Serious	Serious	Low	Low	Low	Low	Moderate	Serious
Tobamidanik & Zabkiewicz ^18^	Low	Low	Low	Low	NI	Low	Low	Low
Morita et al. ^5^	Low	Moderate	Low	Low	Low	Low	Low	Moderate
Neuner et al. ^16^	Low	Moderate	Low	Low	Moderate	Low	Low	Moderate
Poppius et al. ^26^	Moderate	Low	Low	Low	Low	Low	Low	Moderate
Ristkari et al. ^6^	Serious	Low	Low	Low	Moderate	Low	Low	Serious
Saade & Marchand ^22^	Low	Low	Low	Low	Low	Low	Low	Low
Savolainen et al. ^20^	Moderate	Low	Low	Low	Low	Low	Low	Low
Silarova et al. ^4^	Moderate	Moderate	Low	Low	Moderate	Low	Low	Moderate
Thomas et al. ^25^	Low	Low	Low	Low	Moderate	Low	Low	Moderate
Verešová & Gatial ^32^	Serious	Serious	NI	Moderate	NI	Moderate	Moderate	Serious
Vilela & Alisson ^30^	Low	Moderate	Low	Low	NI	Low	Low	Moderate
Von Ah et al. ^23^	Serious	Moderate	Low	Moderate	Serious	Low	Low	Serious
van Loon et al. ^21^	Moderate	Low	Low	Low	Moderate	Low	Low	Moderate
Wainwright et al. ^19^	Low	Low	Low	Low	Low	Low	Low	Low

NI: not informed.

**Table 2:** Certainty of the evidence of the outcomes included in the meta-analysis.

Participants (studies)	Risk of bias	Inconsistency	Indirectness	Imprecision	Publication bias	Overall certainty of evidence	Relative effect (95% CI)
Alcohol							
53,855 (8 nonrandomized studies)	Serious	Serious *	Not serious	Not serious	None	⨁◯◯◯ Very low	OR = 0.7 (0.5; 0.9)
Smoking							
44,494 (11 nonrandomized studies)	Serious	Serious *	Not serious	Not serious	None	⨁◯◯◯ Very low	OR = 0.92 (0.82; 1.01)
Illicit drugs							
4,370 (2 nonrandomized studies)	Serious	Not serious	Not serious	Serious	Publication bias strongly suspected; strong association **	⨁◯◯◯ Very low	OR = 0.31 (0.04; 0.59)

95%CI: 95% confidence interval; OR: odds ratio.

* Inconsistency assessment was based on the dissimilarity of effect estimates;

** Only two studies were included.

### OR for substance use

**Figure 2:**
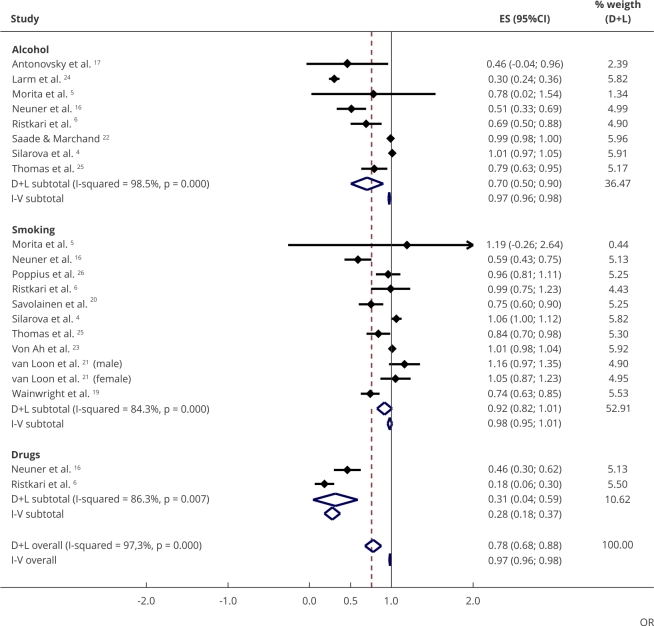
Odds ratios (OR) for sense of coherence (strong) and their effect on substance use type (smoking, alcohol, illicit drugs): 21 studies.

Figure 2 summarizes the results from the 21 studies evaluating the association between SOC and substance use. Individuals with high SOC had a 22% lower odds of using any substance compared with individuals with low SOC (pooled effect: OR = 0.78, 95%CI: 0.68; 0.88, very low degree of certainty). When stratified by substance type, we observed that a strong SOC slightly reduced the odds of smoking (n = 11), but the confidence interval included the reference (pooled effect: OR = 0.92, 95%CI: 0.82; 1.01). Regarding alcohol use (n = 8), individuals with a strong SOC had 30% lower odds of using it (pooled effect: OR = 0.70, 95%CI: 0.50; 0.90, very low degree of certainty). Only two studies analyzed the association with use of illicit drugs, showing a pooled OR of 0.31 (95%CI: 0.04; 0.59).

95%CI: 95% confidence interval; ES: effect size. Note: “D+L” refers to the DerSimonian and Laird method for estimating the aggregate mean effect. “I-V” is an Inverse-Variance approach.

Table 3 presents the subgroup analyses according to characteristics of the analyzed studies. Regarding smoking, studies that evaluated young adults showed a similar association (pooled effect: OR = 0.91, 95%CI: 0.74; 1.09) to those that evaluated older individuals (pooled effect: OR = 0.99, 95%CI: 0.75; 1.23). As for alcohol use, studies that adjusted their estimates for confounding variables reported a weaker association (pooled OR = 0.89, 95%CI: 0.80; 0.98) than those reporting crude estimates (pooled OR = 0.51, 95%CI: 0.22; 0.80). The pooled OR remained similar across studies that evaluated different age groups and continents.

Egger’s test results indicated a trend of small-study effects or publication bias, albeit not statistically significant (p = 0.07). However, we must consider that this analysis is underpowered.

**Table 3:** Sense of coherence (strong) and odds ratios (OR) of substance use: random-effects meta-analyses by subgroup (n = 19).

Subgroup analysis	Smoking				Alcohol			
	Number of estimates	Pooled OR (95%CI)	I-squared (%)	p-value *	Number of estimates	Pooled OR (95%CI)	I-squared (%)	p-value *
Follow-up rate (%)				0.125				0.190
< 50	2	0.81 (0.40; 1.22)	95.8		1	0.51 (0.33; 0.69)	0.0	
50-70	4	1.02 (0.90; 1.14)	67.4		3	0.70 (0.17; 1.23)	99.4	
> 70	6	0.84 (0.72; 0.96)	51.3		4	0.78 (0.52; 1.04)	78.8	
Sample size				0.016				0.599
< 1,000	3	1.02 (0.99; 1.05)	0.0		3	0.82 (0.44; 1.19)	59.7	
1,000-1,999	1	0.84 (0.70; 0.98)	0.0		1	0.79 (0.63; 0.95)	0.0	
≥ 2,000	7	0.88 (0.74; 1.03)	81.7		4	0.62 (0.17; 1.07)	99.3	
Participants age				0.605				0.259
Young	2	0.81 (0.40; 1.22)	95.8		1	0.51 (0.33; 0.69)	0.0	
Adult	6	0.97 (0.83; 1.11)	76.9		6	0.74 (0.51; 0.97)	98.9	
Older adult	1	0.99 (0.75; 1.23)	0.0		1	0.69 (0.50; 0.88)	0.0	
Study design				0.660				0.736
Cross-sectional	6	0.90 (0.78; 1.01)	84.3		5	0.67 (0.24; 1.10)	98.8	
Longitudinal	5	0.95 (0.75; 1.15)	83.3		3	0.74 (0.41; 1.01)	94.5	
Adjustment for confounding				< 0.001				< 0.001
No	5	0.94 (0.82; 1.07)	79.9		3	0.48 (0.17; 0.79)	86.4	
Age	3	0.91 (0.69; 1.13)	82.6		1	0.79 (0.63; 0.95)	0.0	
Age + gender + 1 socioeconomic status variable	1	1.06 (1.00; 1.12)	84.3		1	1.01 (0.97; 1.05)	0.0	
Age + gender + additional substance use	2	0.60 (0.43; 0.76)	0.0		3	0.76 (0.35; 1.17)	92.7	
Continent				0.228				< 0.001
America	1	1.01 (0.98; 1.04)	0.0		5	0.66 (0.28; 1.04)	98.8	
Europe	9	0.90 (0.78; 1.02)	85.9		1	0.99 (0.98; 1.00)	0.0	
Asia	1	1.19 (0.26; 2.64)	0.0		2	0.56 (0.14; 0.97)	0.0	
Categorization of sense of coherence				0.187				0.032
Numerical	2	0.94 (0.78; 1.10)			2	0.91 (0.71; 1.10)	83.3	
Dichotomous	4	1.00 (0.81; 1.18)			2	0.79 (0.27; 1.32)	78.3	
Terciles	2	0.96 (0.81; 1.11)			2	0.60 (0.42; 0.77)	34.3	
Quartiles	2	0.78 (0.39; 1.17)			2	0.91 (0.71; 1.10)	45.0	
Quintiles	1	0.75 (0.60; 0.90)						
Categorization of smoking				0.012				
Smokers (current)/ex and non-smokers	6	0.81 (0.62; 0.99)	89.7					
Smokers (current) and ex/never	5	1.01 (0.98; 1.05)	0.0					
Categorization of alcohol								0.002
Drinkers (current)/ex and non-drinker					4	0.79 (0.51; 1.06)	80.2	
Hazardous alcohol consumption					3	0.53 (0.22; 0.84)	93.9	
Alcohol misuse					1	0.99 (0.98; 1.00)	0.0	
Total	11	0.92 (0.82; 1.01)			8	0.70 (0.50; 0.90)		

95%CI: 95% confidence interval.

* p-value - test of group differences.

### Mean SOC

**Figure 3:**
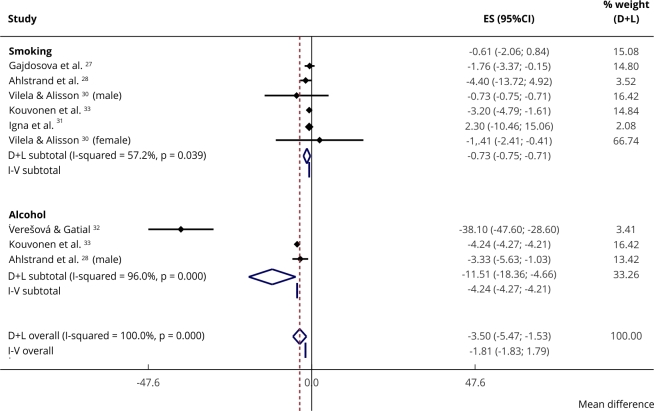
Mean difference in sense of coherence regarding substance use (smoking, alcohol): nine studies.

Seven studies assessed the mean SOC among substance users and non-users assessed using the SOC-13 scale. The pooled mean difference was -3.50 points on the SOC scale (95%CI: -5.47; -1.53) (total score can range from 13 to 65 points) for substance users compared with non-users (Figure 3).

95%CI: 95% confidence interval; ES: effect size. Note: “D+L” refers to the DerSimonian and Laird method for estimating the aggregate mean effect. “I-V” is an Inverse-Variance approach.

### Other measures - regression coefficients

One study estimated the beta coefficient, indicating that a 0.1-point decrease on the SOC scale corresponds to a 1-point increase on the *Alcohol Problem Index* (a Stern index scale).

## Discussion

This systematic review identified 21 studies on the association between SOC and substance use in adults. Our findings indicate a negative association between SOC and substance use, suggesting that individuals with a strong SOC are less likely to use alcohol and illicit drugs.

Regarding confounding control, half of the studies included in the meta-analyses did not adjust their estimates for confounding by socioeconomic status or other variables. As for alcohol use, studies that reported crude estimates showed a stronger association compared with those that controlled for confounding variables, thereby suggesting that confounding overestimated the association between SOC and substance use. Socioeconomic and demographic factors play a crucial role in shaping meaningful experiences that contribute to developing a strong SOC in adulthood ^12^. Socioeconomic status has been positively associated with SOC ^13^ but negatively associated with substance use ^14^ ^,^ ^15^. Consequently, socioeconomic status would overestimate the magnitude of the association between SOC and substance use. As previously described, the magnitude of the association between SOC and alcohol use was weak in those studies that controlled for confounding variables. Thus, further studies evaluating the association of SOC with substance should adjust their estimates to socioeconomic variables.

This study has several strengths, such as the independent literature search conducted by two authors. All studies included collected self-reported data on both substance use and SOC, thus minimizing the occurrence of information bias. Additionally, only studies using validated and standardized instruments to assess SOC were included, thus reducing the possibility of misclassification. One limitation concerns the small number of identified studies measuring issues such as illicit drug use.

Analysis revealed a more consistent association of SOC with alcohol use than with smoking; however, precise measurement of this relation for illicit drugs was hindered by the limited number of studies available. Scarcity of evidence prevents definitive conclusions about the association between SOC and illicit substance use. This research gap suggests that SOC may be more strongly linked to behaviors related to more severe substances like illicit drugs and hazardous alcohol consumption, but confirming this hypothesis would require more comprehensive and specific investigation. Acknowledging this limitation highlights the pressing need for future research focused on understanding the correlation between SOC and use of different types of substances, particularly those considered severe.

Certainty of evidence as assessed by the GRADE system was very low, thus more robust study designs such as randomized clinical trials and longitudinal studies are needed to better understand the impact of a strong SOC on substance use (alcohol, smoking, and illicit drugs). Future research could examine how specific socioeconomic aspects interact with SOC to influence substance use, looking at factors like income, education, and occupation individually. Increasing the certainty of evidence through rigorous study designs is crucial for elaborating effective interventions and public health policies aimed at reducing harmful substance use and promoting overall well-being. Including diverse populations in research, considering ethnicity, culture, and geography, would enhance result generalizability and provide a more comprehensive understanding of how SOC operates across various demographic groups. Moreover, the consistency of our findings with another recent review involving adolescent and young adult populations highlights SOC as a potential protective factor against harmful substance use, reinforcing the clinical and public health relevance of these findings.

Overall, this systematic review and meta-analysis suggest that a strong SOC protects against substance use (alcohol, smoking, and illicit drugs) among adults, regardless of age. As practical implications, these findings suggest that early identification of individuals with low SOC may indicate the need for preventive interventions related to substance use. Recognizing and addressing a diminished SOC early could guide tailored interventions aimed at fortifying resilience and mitigating the risk of substance-related issues, especially in high-risk individuals.

## Supplementary Material


