# Supplementary material for: Sense of coherence and substance use in adults: a systematic review and meta-analysis
Source: Cad Saude Publica. 2024 Sep 20;40(9):e00141323. doi: 10.1590/0102-311XEN141323 (PMC11415044; doi:10.1590/0102-311XEN141323)
Supplement: Supplementary file 1 [file 1678-4464-csp-40-09-EN141323-s.pdf]

SUPPLEMENTARY MATERIAL

Box S1 Prisma checklist.

SECTION AND TOPIC	ITEM #	CHECKLIST ITEM	LOCATION WHERE ITEM IS REPORTED
**TITLE**			
Title	1	Identify the report as a systematic review.	Pg. 1
**ABSTRACT**			
Abstract	2	See the PRISMA 2020 for Abstracts checklist.	Pg. 1
**INTRODUCTION**			
Rationale	3	Describe the rationale for the review in the context of existing knowledge.	Pg. 1
Objectives	4	Provide an explicit statement of the objective(s) or question(s) the review addresses.	Pg. 1
**METHODS**			
Eligibility criteria	5	Specify the inclusion and exclusion criteria for the review and how studies were grouped for the syntheses.	Pg. 2
Information sources	6	Specify all databases, registers, websites, organisations, reference lists and other sources searched or consulted to identify studies. Specify the date when each source was last searched or consulted.	Pg. 2
Search strategy	7	Present the full search strategies for all databases, registers and websites, including any filters and limits used.	Suppl. Material 3
Selection process	8	Specify the methods used to decide whether a study met the inclusion criteria of the review, including how many reviewers screened each record and each report retrieved, whether they worked independently, and if applicable, details of automation tools used in the process.	Pg. 2-3
Data collection process	9	Specify the methods used to collect data from reports, including how many reviewers collected data from each report, whether they worked independently, any processes for obtaining or confirming data from study investigators, and if applicable, details of automation tools used in the process.	Pg. 3
Data items	10a	List and define all outcomes for which data were sought. Specify whether all results that were compatible with each outcome domain in each study were sought (e.g. for all measures, time points, analyses), and if not, the methods used to decide which results to collect.	Pg. 2
	10b	List and define all other variables for which data were sought (e.g. participant and intervention characteristics, funding sources). Describe any assumptions made about any missing or unclear information.	Pg. 3
Study risk of bias assessment	11	Specify the methods used to assess risk of bias in the included studies, including details of the tool(s) used, how many reviewers assessed each study and whether they worked independently, and if applicable, details of automation tools used in the process.	Pg. 3
Effect measures	12	Specify for each outcome the effect measure(s) (e.g. risk ratio, mean difference) used in the synthesis or presentation of results.	Pg. 3-4
Synthesis methods	13a	Describe the processes used to decide which studies were eligible for each synthesis (e.g. tabulating the study intervention characteristics and comparing against the planned groups for each synthesis (item #5)).	Pg. 3-4
	13b	Describe any methods required to prepare the data for	N/A

		presentation or synthesis, such as handling of missing summary statistics, or data conversions.	
	13c	Describe any methods used to tabulate or visually display results of individual studies and syntheses.	Pg. 3
	13d	Describe any methods used to synthesize results and provide a rationale for the choice(s). If meta-analysis was performed, describe the model(s), method(s) to identify the presence and extent of statistical heterogeneity, and software package(s) used.	Pg. 4
	13e	Describe any methods used to explore possible causes of heterogeneity among study results (e.g. subgroup analysis, meta-regression).	Pg. 4
	13f	Describe any sensitivity analyses conducted to assess robustness of the synthesized results.	Pg. 4
Reporting bias assessment	14	Describe any methods used to assess risk of bias due to missing results in a synthesis (arising from reporting biases).	Pg. 4
Certainty assessment	15	Describe any methods used to assess certainty (or confidence) in the body of evidence for an outcome.	Pg. 4
**RESULTS**			
Study selection	16a	Describe the results of the search and selection process, from the number of records identified in the search to the number of studies included in the review, ideally using a flow diagram.	Fig. 1
	16b	Cite studies that might appear to meet the inclusion criteria, but which were excluded, and explain why they were excluded.	Suppl. Material 4
Study characteristics	17	Cite each included study and present its characteristics.	Suppl. Material 5,6
Risk of bias in studies	18	Present assessments of risk of bias for each included study.	Suppl. Material 7
Results of individual studies	19	For all outcomes, present, for each study: (a) summary statistics for each group (where appropriate) and (b) an effect estimate and its precision (e.g. confidence/credible interval), ideally using structured tables or plots.	Suppl. Material 5,6
Results of syntheses	20a	For each synthesis, briefly summarise the characteristics and risk of bias among contributing studies.	Pg. 4
	20b	Present results of all statistical syntheses conducted. If meta-analysis was done, present for each the summary estimate and its precision (e.g. confidence/credible interval) and measures of statistical heterogeneity. If comparing groups, describe the direction of the effect.	Fig. 2
	20c	Present results of all investigations of possible causes of heterogeneity among study results.	Pg. 4
	20d	Present results of all sensitivity analyses conducted to assess the robustness of the synthesized results.	Pg. 4
Reporting biases	21	Present assessments of risk of bias due to missing results (arising from reporting biases) for each synthesis assessed.	Suppl. Material 7
Certainty of evidence	22	Present assessments of certainty (or confidence) in the body of evidence for each outcome assessed.	Suppl. Material 8
**DISCUSSION**			
Discussion	23a	Provide a general interpretation of the results in the context of other evidence.	Pg. 6
	23b	Discuss any limitations of the evidence included in the review.	Pg. 7
	23c	Discuss any limitations of the review processes used.	Pg. 7
	23d	Discuss implications of the results for practice, policy, and future research.	Pg. 7
**OTHER INFORMATION**			
Registration and protocol	24a	Provide registration information for the review, including register name and registration number, or state that the review	Pg. 2

		was not registered.	
	24b	Indicate where the review protocol can be accessed, or state that a protocol was not prepared.	Pg. 2
	24c	Describe and explain any amendments to information provided at registration or in the protocol.	Pg. 3
Support	25	Describe sources of financial or non-financial support for the review, and the role of the funders or sponsors in the review.	Pg. 1
Competing interests	26	Declare any competing interests of review authors.	Pg. 1
Availability of data, code and other materials	27	Report which of the following are publicly available and where they can be found: template data collection forms; data extracted from included studies; data used for all analyses; analytic code; any other materials used in the review.	N/A

Box S2 AMSTAR-2 checklist.

1. Did the research questions and inclusion criteria for the review include the components of PICO?

For Yes:
- ☐ Population
- ☐ Intervention
- ☐ Comparator group
- ☐ Outcome

Optional (recommended)
- Timeframe for follow-up

- Yes
- No

2. Did the report of the review contain an explicit statement that the review methods were established prior to the conduct of the review and did the report justify any significant deviations from the protocol?

For Partial Yes:
The authors state that they had a written protocol or guide that included ALL the following:
- ☐ review question(s)
- ☐ a search strategy
- ☐ inclusion/exclusion criteria
- ☐ a risk of bias assessment

For Yes:
As for partial yes, plus the protocol should be registered and should also have specified:
- ☐ a meta-analysis/synthesis plan, if appropriate, *and*
- ☐ a plan for investigating causes of heterogeneity
- ☐ justification for any deviations from the protocol

- ☐ Yes
- ☐ Partial Yes
- ☐ No

3. Did the review authors explain their selection of the study designs for inclusion in the review?

For Yes, the review should satisfy ONE of the following:
- ☐ *Explanation for* including only RCTs
- ☐ OR *Explanation for* including only NRSI
- ☐ OR *Explanation for* including both RCTs and NRSI

- ☐ Yes
- ☐ No

4. Did the review authors use a comprehensive literature search strategy?

For Partial Yes (all the following):
- ☐ searched at least 2 databases (relevant to research question)
- ☐ provided key word and/or search strategy
- ☐ justified publication restrictions (e.g. language)

For Yes, should also have (all the following):
- ☐ searched the reference lists / bibliographies of included studies
- ☐ searched trial/study registries
- ☐ included/consulted content experts in the field
- ☐ where relevant, searched for grey literature
- ☐ conducted search within 24 months of completion of the review

- ☐ Yes
- ☐ Partial Yes
- ☐ No

5. Did the review authors perform study selection in duplicate?

For Yes, either ONE of the following:
- ☐ at least two reviewers independently agreed on selection of eligible studies and achieved consensus on which studies to include
- ☐ OR two reviewers selected a sample of eligible studies and achieved good agreement (at least 80 percent), with the remainder selected by one reviewer.

- ☐ Yes
- ☐ No

6. Did the review authors perform data extraction in duplicate?

For Yes, either ONE of the following:

- ☐ at least two reviewers achieved consensus on which data to extract from included studies
- ☐ OR two reviewers extracted data from a sample of eligible studies <u>and</u> achieved good agreement (at least 80 percent), with the remainder extracted by one reviewer.

☐ Yes
☐ No

7. Did the review authors provide a list of excluded studies and justify the exclusions?

For Partial Yes:

- ☐ provided a list of all potentially relevant studies that were read in full-text form but excluded from the review

For Yes, must also have:

- ☐ Justified the exclusion from the review of each potentially relevant study

☐ Yes
☐ Partial Yes
☐ No

8. Did the review authors describe the included studies in adequate detail?

For Partial Yes (ALL the following):

- ☐ described populations
- ☐ described interventions
- ☐ described comparators
- ☐ described outcomes
- ☐ described research designs

For Yes, should also have ALL the following:

- ☐ described population in detail
- ☐ described intervention in detail (including doses where relevant)
- ☐ described comparator in detail (including doses where relevant)
- ☐ described study's setting
- ☐ timeframe for follow-up

☐ Yes
☐ Partial Yes
☐ No

9. Did the review authors use a satisfactory technique for assessing the risk of bias (RoB) in individual studies that were included in the review?

RCTs

For Partial Yes, must have assessed RoB from

- ☐ unconcealed allocation, *and*
- ☐ lack of blinding of patients and assessors when assessing outcomes (unnecessary for objective outcomes such as all-cause mortality)

For Yes, must also have assessed RoB from:

- ☐ allocation sequence that was not truly random, *and*
- ☐ selection of the reported result from among multiple measurements or analyses of a specified outcome

☐ Yes
☐ Partial Yes
☐ No
☐ Includes only NRSI

NRSI

For Partial Yes, must have assessed RoB:

- ☐ from confounding, *and*
- ☐ from selection bias

For Yes, must also have assessed RoB:

- ☐ methods used to ascertain exposures and outcomes, *and*
- ☐ selection of the reported result from among multiple measurements or analyses of a specified outcome

☐ Yes
☐ Partial Yes
☐ No
☐ Includes only RCTs

10. Did the review authors report on the sources of funding for the studies included in the review?

For Yes

- ☐ Must have reported on the sources of funding for individual studies included in the review. Note: Reporting that the reviewers looked for this information but it was not reported by study authors also qualifies

○ Yes
○ No

11. If meta-analysis was performed did the review authors use appropriate methods for statistical combination of results?

RCTs

For Yes:

- ☐ The authors justified combining the data in a meta-analysis
  - AND they used an appropriate weighted technique to combine study results and adjusted for heterogeneity if present.
  - AND investigated the causes of any heterogeneity

☐ Yes
☐ No
☐ No meta-analysis conducted

For NRSI

For Yes:

- ☐ The authors justified combining the data in a meta-analysis
  - AND they used an appropriate weighted technique to combine study results, adjusting for heterogeneity if present
  - AND they statistically combined effect estimates from NRSI that were adjusted for confounding, rather than combining raw data, or justified combining raw data when adjusted effect estimates were not available
  - AND they reported separate summary estimates for RCTs and NRSI separately when both were included in the review

☐ Yes
☐ No
☐ No meta-analysis conducted

12. If meta-analysis was performed, did the review authors assess the potential impact of RoB in individual studies on the results of the meta-analysis or other evidence synthesis?

For Yes:

- ☐ included only low risk of bias RCTs
- ☐ OR, if the pooled estimate was based on RCTs and/or NRSI at variable RoB, the authors performed analyses to investigate possible impact of RoB on summary estimates of effect.

☐ Yes
☐ No
☐ No meta-analysis conducted

13. Did the review authors account for RoB in individual studies when interpreting/ discussing the results of the review?

For Yes:

- ☐ included only low risk of bias RCTs
- ☐ OR, if RCTs with moderate or high RoB, or NRSI were included the review provided a discussion of the likely impact of RoB on the results

☐ Yes
☐ No

14. Did the review authors provide a satisfactory explanation for, and discussion of, any heterogeneity observed in the results of the review?

For Yes:

- ☐ There was no significant heterogeneity in the results
- ☐ OR if heterogeneity was present the authors performed an investigation of sources of any heterogeneity in the results and discussed the impact of this on the results of the review

☐ Yes
☐ No

15. If they performed quantitative synthesis did the review authors carry out an adequate investigation of publication bias (small study bias) and discuss its likely impact on the results of the review?

For Yes:

- ☐ performed graphical or statistical tests for publication bias and discussed the likelihood and magnitude of impact of publication bias

☐ Yes
☐ No
☐ No meta-analysis conducted

16. Did the review authors report any potential sources of conflict of interest, including any funding they received for conducting the review?

For Yes:	
☐ The authors reported no competing interests OR<br>☐ The authors described their funding sources and how they managed potential conflicts of interest	☐ Yes<br>☐ No

Table S1 Database search strategy on substance use/health risk behaviors.

<table>
<tr><th>Database</th><th>Terms used</th><th>Identified Articles</th></tr>
<tr><td>MEDLINE</td><td rowspan="3">("sense of coherence" OR "salutogenesis") AND ("smoking" OR "cigarette smoking" OR "tobacco" OR "Tobacco Use Disorder" OR “Alcohol” OR "Alcoholism" OR "alcohol drinking" OR “substance use” OR "Substance-Related Disorders" OR "Substance Abuse" OR "Illicit Drugs" OR "cocaine" OR "crack" OR "cannabis" OR "Amphetamine" OR "narcotic")</td><td>186</td></tr>
<tr><td>Web of Science</td><td>180</td></tr>
<tr><td>PsyInfo</td><td>168</td></tr>
<tr><td>Lilacs</td><td>("senso de coerência" OR "salutogênese") AND ("fumo" OR "álcool" OR "tabaco" OR "alcoolismo" OR “abuso de substância” OR “uso de substância” OR “drogas ilícitas” OR “crack” OR “maconha” OR “cocaína” OR “narcótico”)</td><td>32</td></tr>
<tr><td>Total</td><td></td><td>566</td></tr>
</table>

Box S3 References that were excluded after their full text was reviewed and the reasons for exclusion.

Excluded references	Reasons
Kerr WC, Ye Y. Relationship of life-course drinking patterns to diabetes, heart problems, and hypertension among those 40 and older in the 2005 U.S. National Alcohol Survey. J Stud Alcohol Drugs. 2010;71(4):515-525. doi:10.15288/jsad.2010.71.515	Data on the association between sense of coherence and substance use not presented.
Shechory Bitton M, Noach HB. Psychological factors and the use of psychoactive substances in relation to sexual orientation: A study on Israeli young adults. Curr Psychol. Published online May 6, 2022. doi:10.1007/s12144-022-03189-6	Data on the association between sense of coherence and substance use not presented.
Allison KR, Adlaf EM, Ialomiteanu A, Rehm J. Predictors of health risk behaviours among young adults: analysis of the National Population Health Survey. Can J Public Health. 1999;90(2):85-89. doi:10.1007/BF03404107	Data on the association between sense of coherence and substance use not presented.
Arghabaei, Mohammad et al. "The Role of Family Emotional Atmosphere, Sense of Coherence, and Affects in the Prediction of Tendency Toward Substance Use Among University Students." Iranian Journal of Psychiatry & Clinical Psychology (2018): n. pag.	Data on the association between sense of coherence and substance use not presented.
Moutinho LSM, Mendes AMdOC, Lopes MJ. Alcohol consumption and the sense of coherence in young people in educational training. SMAD Revista eletrônica saúde mental álcool e drogas. 2015;11:208-16.	Data on the association between sense of coherence and substance use not presented.
Franke A. Substanzkonsum von Frauen - Ergebnisse einer salutogenetischen Untersuchung [Consumption of alcohol and medicaments of women--results of a salutogenetic inquiry]. Zentralbl Gynakol. 2002;124(6):331-335. doi:10.1055/s-2002-34745	Data on the association between sense of coherence and substance use not presented.
Nyamathi AM. Relationship of resources to emotional distress, somatic complaints, and high-risk behaviors in drug recovery and homeless minority women. Res Nurs Health. 1991;14(4):269-277. doi:10.1002/nur.4770140405	Data on the association between sense of coherence and substance use not presented.
Adorni R, Zanatta F, D'Addario M, et al. Health-Related Lifestyle Profiles in Healthy Adults: Associations with Sociodemographic Indicators, Dispositional Optimism, and Sense of Coherence. Nutrients. 2021;13(11):3778. Published 2021 Oct 25. doi:10.3390/nu13113778	Data on the association between sense of coherence and substance use not presented
Chen G. Gender differences in crime, drug addiction, abstinence, personality characteristics, and negative emotions. J Psychoactive Drugs. 2009 Sep;41(3):255-66. doi: 10.1080/02791072.2009.10400536. PMID: 19999679.	Sample of users – without a comparison group.
Badura K, Gorczyca P, Tomalczyk E, Matysiakiewicz J. Ocena poczucia koherencji u pacjentów z zespołem zalezności alkoholowej--doniesienie wstepne [Estimation of a sense of coherence in patients with alcoholic dependence syndrome--introductory report]. Wiad Lek. 2000;53(9-10):488-492.	Sample of users – without a comparison group.
Nomoto M, Hara A, Kikuchi K. Effects of long-time commuting and long-hour working on lifestyle and mental health among school teachers in tokyo, JAPAN. J Hum Ergol (Tokyo). 2015;44(1):1-9.	Sample of users – without a comparison group.
de Oliveira Miranda L, Neiva da Silva A, Pereira da Cunha I, Luiz Mialhe F, Laura Cortellazzi K, Rodrigues Lacerda V. Sense of coherence and oral health of users of psychoactive substances. Journal of Substance Use. 2021;26(6):639-44.	Sample of users – without a comparison group.
Lundqvist T. Chronic cannabis use and the sense of coherence. Life Sciences. 1995;56(23):2145-50.	Sample of users – without a comparison group.
Gila Chen. Gender differences in sense of coherence, perceived social support, and negative emotions among drug-abstinent israeli inmates. Int J Offender Ther Comp Criminol. 2010;54(6):937-958. doi:10.1177/0306624X09343185	Sample of users – without a comparison group.
Riera-Sampol A, Bennasar-Veny M, Tauler P, Nafría M, Colom M, Aguilo A. Association between Depression, Lifestyles, Sleep Quality and Sense of Coherence in a Population with Cardiovascular Risk. Nutrients. 2021;13(2):585. Published 2021 Feb 10. doi:10.3390/nu13020585	Outcomes and exposure that were not of interest
Malinauskiene V, Leisyte P, Romualdas M, Kirtiklyte K. Associations between self-rated health and psychosocial conditions, lifestyle factors and health resources among hospital nurses in Lithuania. J Adv Nurs. 2011;67(11):2383-2393. doi:10.1111/j.1365-2648.2011.05685.x	Outcomes and exposure that were not of interest
Binkowska-Bury M, Kruk W, Szymanska J, Marc M, Penar-Zadarko B, Wdowiak L. Psychosocial factors and health-related behavior among students from South-East Poland. Ann Agric Environ Med. 2010;17(1):107-113.	Outcomes and exposure that were not of interest
Vyas D, Patel M, Sharma A, Chhabra KG, Gupta A, Mundra R. Impact of self-efficacy and sense of coherence on tobacco cessation motivation and readiness among slum dwellers in Ajmer city during COVID-19 health emergency. J Family Med Prim Care. 2022;11(5):1867-1875. doi:10.4103/jfmpc.jfmpc_1821_21	Outcomes and exposure that were not of interest
Sarit S, Rajesh G, Eriksson M, Pai M. Impact of Sense of Coherence on Oral Health Behaviour and Perceived Stress among a Rural Population in South India-An Exploratory Study. Journal of Clinical and Diagnostic Research.	Outcomes and exposure that were not of interest

2020;14:ZC01-ZC4.	
Abramsohn Y, Peles E, Potik D, Schreiber S, Adelson M. Sense of coherence as a stable predictor for methadone maintenance treatment (MMT) outcome. J Psychoactive Drugs. 2009;41(3):249-253. doi:10.1080/02791072.2009.10400535	Outcomes and exposure that were not of interest
Stankūnas M, Kalediene R, Starkuviene S. Sense of coherence and its associations with psychosocial health: results of survey of the unemployed in Kaunas. Medicina (Kaunas). 2009;45(10):807-13.	Outcomes and exposure that were not of interest
Escobar-Castellanos, Blanca, Cid-Henríquez, Patricia, Juvinyà-Canal, Dolors, & Sáez-Carrillo, Katia. (2019). Estilo de vida promotor de salud y sentido de coherencia en adultos jóvenes universitarios. Hacia la Promoción de la Salud, 24(2), 107-122. https://doi.org/10.17151/hpsal.2019.24.2.9	Outcomes and exposure that were not of interest
Kuuppelomäki M, Utriainen P. A 3 year follow-up study of health care students' sense of coherence and related smoking, drinking and physical exercise factors. Int J Nurs Stud. 2003;40(4):383-388. doi:10.1016/s0020-7489(02)00103-7	Outcomes and exposure that were not of interest
Ogawa Y, Nakamura H, Nagase H, Ogino K, Ooshita Y, Tsukahara S. Nihon Eiseigaku Zasshi. 2001;55(4):597-606. doi:10.1265/jjh.55.597	Outcomes and exposure that were not of interest
Peker K, Bermek G, Uysal O. Factors related to sense of coherence among dental students at Istanbul University. J Dent Educ. 2012;76(6):774-782.	Outcomes and exposure that were not of interest
Packard CJ, Cavanagh J, McLean JS, et al. Interaction of personality traits with social deprivation in determining mental wellbeing and health behaviours. J Public Health (Oxf). 2012;34(4):615-624. doi:10.1093/pubmed/fds030	Outcomes and exposure that were not of interest
Binkowska-Bury M, Marć M, Górajek-Jóźwik J. Poczucie koherencji i wybrane zachowania ryzykowne wśród studentów. Annales Umcs, Medicina. 2008;63:19-26.	Outcomes and exposure that were not of interest
Giotakos O. Suicidal ideation, substance use, and sense of coherence in Greek male conscripts. Mil Med. 2003;168(6):447-450.	Outcomes and exposure that were not of interest
